# Diagnostic Utility of Thoracic Radiography and Abdominal Ultrasonography in Canine Immune-Mediated Polyarthritis: 77 Cases

**DOI:** 10.3390/ani14040534

**Published:** 2024-02-06

**Authors:** Julia W. Y. Tang, Anna Saiz, Alina Vulpe, Ariadna Ribas Latre, Rita Furtado, Mayank Seth, Ferran Valls Sanchez

**Affiliations:** 1 DWR Veterinary Specialists, Station Farm, London Road, Six Mile Bottom, Cambridgeshire CB8 0UH, UKalina18vulpe@gmail.com (A.V.);; 2 Hospital de Referencia Veterios, 28022 Madrid, Spain; 3 KGS Veterinary Services Limited, Saffron Walden, Essex CB11 3GP, UK; mseth69@gmail.com; 4 FVSmedicine Limited, Cambourne, Cambridgeshire CB23 6DJ, UK

**Keywords:** immune-mediated polyarthritis, diagnostic utility, thoracic radiography, abdominal ultrasonography

## Abstract

**Simple Summary:**

Immune-mediated polyarthritis (IMPA) is the most common disease affecting multiple joints in dogs. This study aimed to assess the utility of thoracic radiography and abdominal ultrasonography in canine IMPA and to assess whether abnormal findings influenced the overall case management of the patient at the time of diagnosis. Seventy-seven dogs were included. Thoracic radiography was considered not useful in the overall case management at the time of IMPA diagnosis in 70 cases, whilst abdominal ultrasonography was considered not useful in 57. Therefore, in the majority of the cases in this study, thoracic radiography and abdominal ultrasonography were considered not useful towards the overall case management at the time of initial diagnosis of IMPA.

**Abstract:**

Thoracic radiography and abdominal ultrasonography are part of standard diagnostic investigations in cases of canine immune-mediated polyarthritis (IMPA). However, the clinical importance of thoracic and abdominal imaging towards the management of canine IMPA currently remains unknown. The primary aim of this study was to describe the findings documented on thoracic radiography and abdominal ultrasonography in dogs diagnosed with IMPA, and to evaluate the diagnostic utility of thoracic radiography and abdominal ultrasonography in the initial approach and management of these cases. Seventy-seven dogs diagnosed with IMPA who underwent thoracic radiography and abdominal ultrasonography at a single referral hospital between 2008 and 2022 were included. The diagnostic imaging studies of these 77 dogs were reviewed by one blinded board-certified diagnostic imaging specialist for quality assurance. The medical records, including the diagnostic imaging reports of these dogs, were then reviewed by three blinded board-certified internal medicine specialists. Using a modified version of a previous question and scoring system, the three internal medicine specialists then generated an answer for the overall diagnostic utility and a diagnostic utility score for thoracic radiography and abdominal ultrasonography for each case. The abnormal findings identified in radiography and ultrasonography were described. In the cases where the findings were considered significant enough to immediately affect the case management, the results of the further investigations that were subsequently performed were also described. No abnormalities were detected in thoracic radiography for 30 cases, and none were detected in abdominal ultrasound for 6. The majority of the internists considered thoracic radiography to be not useful in the overall case management at the time of IMPA diagnosis in 70 cases, and considered abdominal ultrasonography to be not useful in the overall case management in 57 cases. The majority of the internists agreed on the utility of thoracic radiography in 95% of the cases, and in 61% of the cases for abdominal ultrasonography. The most common finding in the thoracic radiography was a mild bronchial pulmonary pattern, and the most common in the abdominal ultrasonography was mild lymphadenomegaly. Therefore, although thoracic radiography and abdominal ultrasonography identified numerous abnormal findings in this population of dogs, in the majority of the cases, the findings were deemed not useful towards the overall case management at the time of the initial diagnosis of IMPA. Thus, the use of thoracic radiography and abdominal ultrasonography should be taken into careful consideration when considering initial diagnostic investigations for canine IMPA.

## 1. Introduction

Immune-mediated polyarthritis (IMPA) is the most common polyarthropathy in dogs [1]. The prognosis is fair to good, with reported mortality rates ranging from 15 to 24% [2].

The postulated aetiology is immune complex deposition within the synovial membrane, resulting in the inflammation of the synovium [3,4,5]. Clinical signs include musculoskeletal abnormalities such as lameness and joint swelling, though systemic signs may also be present, such as lethargy, inappetence, and pyrexia [3,5]. In some cases, only systemic clinical signs may be reported, and up to 25% of dogs with IMPA are presented due to non-specific signs of systemic illness [3]. IMPA most commonly leads to non-erosive arthropathy. The aetiology for erosive IMPA remains elusive [4,6,7], and the proportion of dogs with IMPA found to have erosive bone lesions on radiography has been reported between 1.9 to 16% in previous studies [3].

The diagnosis of canine IMPA centres classically on the cytological assessment of synovial fluid, and namely the presence of inflammation and non-degenerate leukocytes in synovial fluid from two or more joints [3,5,8]. Canine IMPA can then be classified into erosive or non-erosive, in accordance with the presence of erosive bony lesions on radiography. The further classification of a non-erosive IMPA currently entails investigations to document any potential associative disease. Thus, canine non-erosive IMPA can be subsequently classified into four types (Table 1), with type I (idiopathic) IMPA considered the most common form in dogs.

Another system of classification divides polyarthropathies into infectious, reactive, and primary (idiopathic immune-mediated) categories, with the further subdivision of primary IMPA into erosive and non-erosive [3]. Non-erosive primary IMPA is then further divided into systemic lupus erythematosus, breed-associated (Shar-Pei and Akita Inu), and idiopathic, whilst erosive primary IMPA is divided into breed-associated (Greyhound), and idiopathic (rheumatoid arthritis). In summary, investigations aim to conclude the presence/absence of an associated disease which may play a role as a trigger for IMPA. However, clinically, it remains challenging to distinguish between a true trigger versus a comorbidity in dogs with IMPA.

Diagnostic investigations for suspected IMPA in dogs generally include haematology, serum biochemistry, urine analysis, vector-borne infectious disease testing (based on geographical location and patient travel history), and thoracic and abdominal imaging to assess for potential associative disease and triggers. Such investigations are a mainstay in referral veterinary centres. However, the access to diagnostic imaging and financial costs may impede such diagnostics for certain patients, and in general practice. In addition, whilst the use of bicavitary imaging has long been recommended in the initial work-up of suspected immune-mediated disease in small animals (including cases of pyrexia of unknown origin) [9,10], the utility and importance of thoracic and abdominal imaging for canine IMPA currently remains unknown.

A study by Stull et al. [1] investigated 83 dogs with immune-mediated polyarthritis, where 56% and 54% of the study population underwent thoracic radiography and abdominal ultrasonography, respectively. The study noted that, in their population of dogs with breed-associated IMPA, reactive IMPA, idiopathic non-erosive IMPA, and systemic lupus erythematosus, no specific findings were documented on the thoracic and abdominal imaging. Nonetheless, the medical records were reviewed by a single author and the clinical relevance of any findings were not scored. Clements et al. [11] also documented that results of thoracic, abdominal, and joint radiography (apart from soft tissue changes), and abdominal ultrasonography were unremarkable in most dogs with type I IMPA. However, only 20 dogs underwent thoracic radiography and 11 dogs underwent abdominal ultrasonography in the study. In addition, another study of 166 dogs by Keyserling et al. [12] revealed that the majority of the dogs who underwent screening thoracic radiographs for non-cardiopulmonary disease did not have their clinical plan changed as a consequence of the detection of any abnormalities on thoracic radiography. In light of these, it was of interest to investigate and describe the relevance and importance of diagnostic imaging findings in a larger sample population of dogs with IMPA, and for the imaging findings to be reviewed by a collective group of internal medicine specialists.

The primary aim of this study was therefore to describe changes documented on thoracic radiography and abdominal ultrasonography in dogs diagnosed with IMPA, and the subsequent utility of these two imaging modalities in the management of IMPA at the time of diagnosis. The secondary aim was to assess the agreement between internal medicine specialists regarding the importance of the abnormal imaging findings, and the clinical utility of the two imaging modalities. The third aim was to describe the presence of erosive changes when joint radiography was performed. Our hypothesis was that common imaging findings would exist but that in the majority of cases, these findings would not to be considered an associated disease or trigger for IMPA, nor lead to any additional diagnostic investigations or therapeutic interventions in the patient. We also hypothesized that erosive changes would be present in the minority of the joints radiographed.

## 2. Materials and Methods

This was a retrospective descriptive study. Medical records of a single veterinary referral centre in the United Kingdom (UK) were reviewed between the years 2008 and 2022. Inclusion criteria were dogs with a confirmed diagnosis of IMPA (at least 2 or more joints with cytological evidence of neutrophilic inflammation in synovial fluid) for which thoracic radiography (at least 2 orthogonal views) and abdominal ultrasonography had been performed within 48 h of arthrocentesis.

When musculoskeletal radiographs were acquired, these were also assessed for the presence or absence of erosive changes. Signalment of each case (age, sex, breed), body weight, and reported clinical signs at initial presentation were also recorded. All cases included in this study had baseline haematology, serum biochemistry, vector-borne infectious disease serology testing based on geographical location in the UK (IDEXX SNAP^®^ 4Dx^®^, US IDEXX Laboratories, Inc. (Westbrook, ME, USA) for *Ehrlichia canis* antibodies, *Ehrlichia ewingii* antibodies, *Borrelia burgdorferi* sensu latu antibodies, *Anaplasma phagocytophilium* antibodies, *Anaplasma platys* antibodies, and *Dirofilaria immitis* antigen), and urine analysis (including sediment examination via a voided sample or by cystocentesis) performed at initial presentation regardless of presenting clinical signs and diagnostic imaging findings.

Arthrocentesis had been performed under sedation or anaesthesia, at the attending clinician’s discretion, and by either a board-certified internal medicine specialist, or an internal medicine resident under supervision. Radiographs had been evaluated and abdominal ultrasound performed by either a board-certified diagnostic imager, or a diagnostic imaging resident under supervision. Synovial fluid cytology had been evaluated by either a board-certified clinical pathologist, or clinical pathology resident under supervision.

The abnormal imaging findings in both thoracic radiography and abdominal ultrasonography were described for the study population [13,14,15,16,17]. The number of dogs who underwent joint radiography was also described, as well as the type and number of joints radiographed, and the number of cases where erosive bone changes [18] were documented.

One blinded board-certified specialist in veterinary diagnostic imaging evaluated the imaging studies from all included cases for quality assurance purposes. Three blinded board-certified specialists in small animal internal medicine (with no involvement in the included cases) then assessed all available radiography and ultrasonography reports and evaluated the clinical utility of these imaging modalities using a modified version of a previous question and scoring system [19]. The overall case management for the purpose of this study was defined as any intervention (diagnostic tests or medical treatment) performed or adjusted during the initial presentation and time of IMPA diagnosis of the patient. Time of initial presentation and diagnosis was defined in this study as ‘within 48 h of IMPA diagnosis’ for a case. The modified clinical utility scoring system consisted of the following two questions:

Question 1—Were the imaging findings considered useful or not useful in the overall case management, whether related or unrelated to the patient’s IMPA? The 2 possible answers were ‘useful’ or ‘not useful’. Imaging findings were considered ‘useful’ if an intervention (alteration in case management) was made as a consequence of abnormal findings on radiography or ultrasonography at the time of initial IMPA diagnosis.
Question 2—Were there any abnormal imaging findings, and if so, were they significant and did they impact the overall case management at time of initial IMPA diagnosis, or did they impact future management? The 4 possible scores were as follows:
  Score 1—No abnormal imaging findings documented.
  Score 2—Abnormal imaging findings were documented, but not considered clinically relevant. For example, age-related changes that were considered incidental findings.
  Score 3—Abnormal imaging findings were documented, but these findings led to a diagnostic and/or therapeutic recommendation for the future, and thus interventions were not immediately applied at the time of the initial IMPA diagnosis. For example, ultrasonographic evidence of chronic kidney disease that led to recommendations for future monitoring of renal biochemistry values.
  Score 4—Abnormal imaging findings were documented, and these findings led to immediate performance of additional diagnostic investigations and/or immediate adjustments to therapeutic management of the patient at time of initial IMPA diagnosis. For example, if abnormal imaging findings prompted the performance of additional diagnostic tests such as fine needle aspirates.

These questions were answered for thoracic radiography and abdominal ultrasonography independently, and the level of agreement between the internal medicine clinicians was calculated for questions 1 and 2. The purpose of question 1 was to assess if the imaging modality revealed any significant findings that affected the overall management of the patient either during its immediate presentation for IMPA, or in the future (relating to IMPA or any other condition identified). A ‘full agreement’ was made if all 3 internists gave the same answer, and ‘partial agreement’ was made if one internist did not agree. For cases of full agreement, the number of cases for each answer (useful/not useful) was described.

The purpose of question 2 was to assess more specifically what the clinical utility of each imaging modality was. The question aimed to answer if no abnormal findings were present, if abnormal imaging findings were present but not considered clinically relevant, if abnormal findings were present and led to a diagnostic and/or therapeutic recommendation for the future (i.e., not performed at time of initial IMPA diagnosis), or if any abnormal findings were present and immediately impacted the initial diagnostic tests and/or treatment plans for the patient at time of IMPA diagnosis. A ‘full agreement’ was made if all 3 internists gave the same score, ‘partial agreement’ if 2 out of the 3 internists gave the same score, and ‘full disagreement’ if each internist gave a different score. For cases of full agreement and partial agreement, the number of cases for each score was described. For cases of ‘partial agreement’, the score given by the majority (2 out of the 3 internists), was described. The frequency of scores were also described for both full and partial agreement. If there was a full agreement for a score of 4, or partial agreement with a majority score of 4, where available, the results of any further diagnostic investigations such as fine needle aspirates (FNAs) and bronchoalveolar lavage (BAL) performed in these patients were described. For cases with full disagreement in question 2, and where at least 1 internist had given a score of 4, the abnormal imaging findings that caused the different scoring were described. The impact of the most common imaging findings in both thoracic radiography and abdominal ultrasonography on question 2 were also described.

## 3. Results

### 3.1. Study Population

One hundred and seven dogs were diagnosed with immune-mediated polyarthritis at the single veterinary referral centre during the study period. Thirty dogs were excluded due to insufficient diagnostic imaging records where either thoracic radiography, abdominal ultrasonography, or both modalities were not performed at initial presentation. Seventy-seven dogs therefore met the inclusion criteria and were included in the study analysis. Eleven dogs were female entire, 28 were female spayed, 18 were male entire, and 20 were male neutered. The mean age at presentation was 54 months (median 55 months, range 2 to 127 months), and the mean body weight at presentation was 20.73 kg (median 19.45 kg, range 4.10 to 55.10 kg).

The most common breed in this population was crossbreeds (*n* = 15), followed by Cocker Spaniel (*n* = 8), and then Labrador Retriever (*n* = 5). The other breeds identified were Boxer (*n* = 4), Golden Retriever (*n* = 4), Jack Russell Terrier (*n* = 4), Border Terrier (*n* = 3), German Shepherd (*n* = 3), and two cases each of Dachshund, Fox Terrier, Miniature Schnauzer, Rhodesian Ridgeback, Springer Spaniel and Staffordshire Bull Terrier, with one case each of Airedale Terrier, Alaskan Malamute, Australian Shepherd, Bearded Collie, Bernese Mountain Dog, Bichon Frise, Border Terrier, Cairn Terrier, Cane Corso Italiano, Flat-Coated Retriever, German Short Haired Pointer, Great Dane, Miniature Dachshund, Miniature Poodle, Pomeranian, Standard Poodle, Weimaraner, West Highland White Terrier, and Whippet.

The reported clinical signs at initial presentation are displayed in Table 2, with the most common clinical sign being lameness, followed by joint swelling and pyrexia.

### 3.2. Thoracic Radiography

#### 3.2.1. Findings in Thoracic Radiography

Thoracic radiographic findings identified in this population are listed in Table 3, with the most common findings being a mild bronchointerstitial pulmonary pattern (19% of the total study population), followed by a mild interstitial pattern (12%) and a mild bronchial pattern (10%).

#### 3.2.2. Overall Diagnostic Utility (ODU)

For the majority of the internists, thoracic radiography was considered not useful for the overall case management at the time of IMPA diagnosis in 70/77 cases (91%, Table 4). Full agreement for radiographic ODU was made by the three internists in 73/77 cases (95%). Additionally, in the majority of the cases where thoracic radiography ODU was in full agreement, the radiographs were found to not affect the overall management of the patient at the time of IMPA diagnosis (68/73 cases [93%]).

#### 3.2.3. Diagnostic Utility Score (DUS)

Thoracic radiography revealed no abnormalities in 30/77 cases (39%, Table 5). In the cases of full agreement for thoracic radiography, the most common scores given were 1 and 2. This conveyed that in the majority of the cases, thoracic radiography either revealed no abnormalities, or revealed abnormal findings not considered by the internists to be clinically relevant to the patient. Full agreement for DUS was made in the majority of the cases (65/77 [84%]) for thoracic radiography, with no cases of full disagreement (Table 5). In five cases, full agreement for a score of 4 was given for thoracic radiography. When partial agreement was made in the thoracic radiographs, the most common score given by the majority of the internists was a score of 2. Overall, abnormal findings on thoracic radiography were considered by the majority of the internists to be clinically irrelevant at the time of IMPA diagnosis (38/47 cases [81%]).

#### 3.2.4. Further Diagnostic Tests and Treatments following Thoracic Radiography Findings

When assessing thoracic radiography, six cases received a majority score of 4 (8% of the total study population). There was full agreement made in five of these cases, and partial agreement in one. In this one case of partial agreement, two internists gave a score of 4, whilst the third gave a score of 3. Of note, none of these six cases presented with respiratory clinical signs.

One dog out of these six did not undergo further diagnostic investigations in actuality, but received immediate adjustment to its case management based solely on abnormal thoracic radiographic findings, and without further investigations, in the form of antimicrobial therapy and reduced corticosteroid dosing due to concerns of aspiration pneumonia. Although this patient had not initially presented with respiratory signs, the attending clinician did consider the aspiration pneumonia a potential associative disease process for its IMPA (type II).

**Figure 1:**
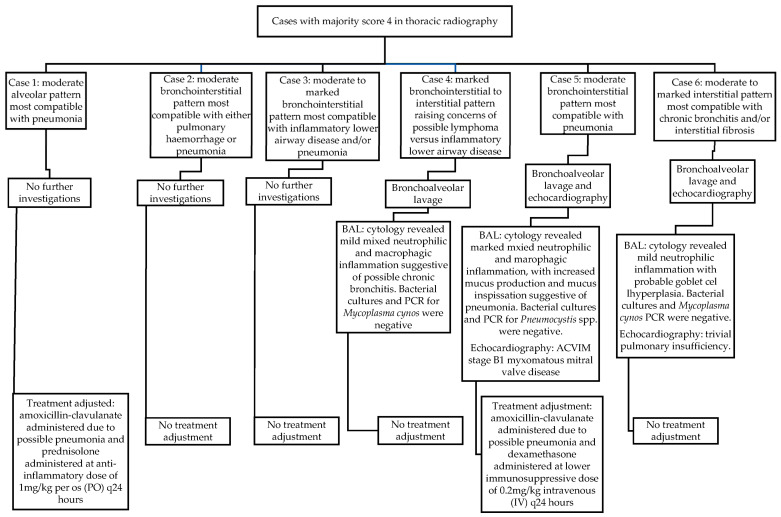
Radiographic findings, further diagnostic investigations, and treatment adjustments in cases scored as 4 in thoracic radiography by the majority of internists.

Three of these six cases (3/6 [50%]) underwent further diagnostic investigations (4% of the total study population). Of these three, only one dog eventually received alterations to its overall case management at the time of IMPA diagnosis as a consequence of further results. In this one case (Figure 1), the BAL findings were compatible with pneumonia and/or acute respiratory distress syndrome (ARDS). This same dog was also diagnosed with American College of Veterinary Internal Medicine (ACVIM) stage B1 myxomatous mitral valve disease (MMVD) following echocardiography. Unfortunately, the patient went on to develop respiratory distress in hospital, at which point a possible pulmonary thromboembolism was suspected (alongside pneumonia and possible ARDS), following which the patient suffered a cardiopulmonary arrest and died in hospital.

Therefore, of the six cases that received a majority score of 4 for thoracic radiography, only two cases (33%) received alterations to their overall case management at the time of IMPA diagnosis (3% of the total study population). The radiographic findings in these six cases that were considered significant enough to receive a majority score of 4, and results of subsequent investigations that occurred at the time of presentation, are displayed in Figure 1.

The most common abnormal thoracic radiography findings (mild bronchointerstitial pulmonary pattern, mild bronchial pattern, and mild interstitial pattern, Table 3) were not considered useful for the overall case management at the time of diagnosis of IMPA for any case, by all three internists.

### 3.3. Abdominal Ultrasonography

#### 3.3.1. Findings in Abdominal Ultrasonography

The abdominal ultrasound findings documented in this population are listed in Table 6, with the most common findings being mild lymphadenomegaly (56% of the cases), followed by peritoneal effusion (14%) and splenomegaly (14%).

#### 3.3.2. Overall Diagnostic Utility (ODU)

For the majority of the internists, abdominal ultrasonography was considered not useful for the overall case management at the time of IMPA diagnosis in 57/77 cases (74%, Table 7). Full agreement for ultrasonographic ODU was made in 47/77 cases (61%). In the majority of the cases where abdominal ultrasonography ODU was in full agreement, the ultrasound was found to not affect the overall case management at the time of IMPA diagnosis (43/47 cases [91%]).

#### 3.3.3. Diagnostic Utility Score (DUS)

Abdominal ultrasound revealed no abnormalities in 6/77 cases (8%, Table 8). When full agreement was made in the abdominal ultrasonography, the most common score given was 2. When partial agreement was made, the most common score given by the majority was also a score of 2. Interestingly, in three cases of partial agreement, the difference was between scores of 2 and 4. In these cases, the significant findings that led to an internist, or internists, giving a score of 4 were mild bilateral adrenomegaly in one dog, hepatomegaly with mild uniform increased echogenicity in the second, and moderate lymphadenomegaly with moderate splenomegaly and moderate hepatomegaly in the third dog. Overall, in the majority of the cases, abnormal findings on abdominal ultrasonography were considered not clinically relevant (45/71 cases [63%]).

#### 3.3.4. Full Disagreement in Diagnostic Utility Scores

In the two cases where full disagreement was made on abdominal ultrasonography, the three different scores given were 1, 2, and 3 in each case. For the first case, two well-defined hypoechoic splenic nodules (0.55 cm and 0.58 cm diameter) and a polypoid wall lesion on the right side of the urinary bladder (compatible with recent cystotomy site) were documented. In the second case, cystic endometrial hyperplasia, and a small volume of fluid in the uterine lumen were identified.

#### 3.3.5. Further Diagnostic Tests and Treatment Interventions following Abdominal Ultrasonography Findings

**Figure 2:**
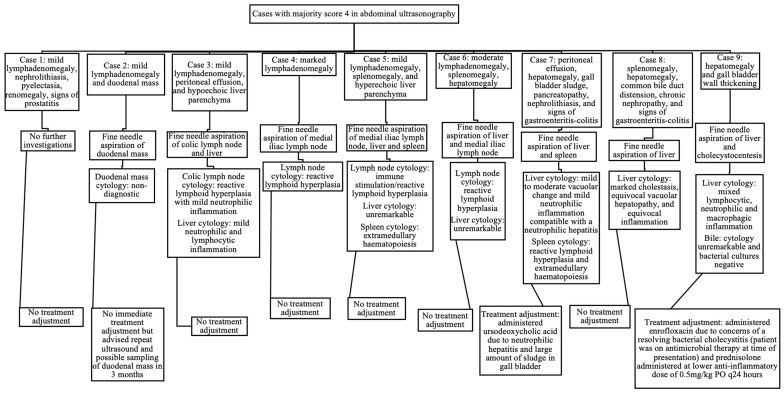
Ultrasonographic findings, further diagnostic investigations, and treatment adjustments in cases scored as 4 in abdominal ultrasonography by the majority of internists.

When assessing abdominal ultrasonography, nine cases received a majority score of 4 (12% of the total study population). There was full agreement in two cases, and partial agreement in the other seven. In these seven cases of partial agreement, there were five cases where the majority (two internists) gave a score of 4 whilst the minority (third internist) gave a score of 3, and there were two cases where the majority gave a score of 4 whilst the minority gave a score of 2. Eight of the nine cases (89%) with a majority score of 4 on ultrasound underwent further diagnostic tests at the time of presentation (10% of the total study population). The ultrasonographic findings in these nine cases that were considered significant enough to receive a majority score of 4, and the results of subsequent investigations that occurred at the time of presentation, are displayed in Figure 2. Of the nine cases that received a majority score of 4 for abdominal ultrasonography, only two cases (22%) received alterations to their overall case management at the time of IMPA diagnosis (3% of the total study population).

However, in one case with a duodenal mass, the cytology results were non-diagnostic and led to recommendation for future monitoring. Therefore, of the nine cases that received a majority score of 4 for abdominal ultrasonography, two cases (25%) had findings significant enough to immediately affect their overall case management at the time of IMPA diagnosis (3% of the total study population).

Of the cases with documented lymphadenomegaly (Table 6), 4/48 cases (8%; two with mild lymphadenomegaly, one with moderate, and one with marked) underwent an FNA of the enlarged lymph node identified. Of the 11 cases with peritoneal effusion identified, none underwent sampling, as the volume of the effusion was considered too scant to sample in all the cases. Of the 11 cases with splenomegaly, only one case (9%) underwent an FNA of the spleen. The eventual cytology results from all the lymph nodes and spleens aspirated did not reveal any significant findings, and thus, did not affect further case management.

### 3.4. Musculoskeletal Radiographs

Of the 77 dogs included in this study, 45 (58%) underwent radiographs of joints to a varying degree. The most common joint radiographed was the carpi (45/45 [100%]), followed by the tarsi (42/45, [93%]), then the stifles (28/45 [62%]), then the elbows (17/45 [38%]), and only one dog underwent radiographs of the hips (2%). The mean number of joints radiographed was three (a range of one to four). Erosive bone changes were reported in only 1 (2%) of these 45 cases.

## 4. Discussion

This study has documented numerous common findings on thoracic radiography and abdominal ultrasonography at the time of diagnosis of IMPA in dogs. The most common radiographic findings were a mild bronchointerstitial pulmonary pattern, mild bronchial pulmonary pattern, and mild interstitial pulmonary pattern, whilst the most common abdominal ultrasonographic findings were mild lymphadenomegaly, peritoneal effusion, and splenomegaly. Overall, both thoracic radiography and abdominal ultrasonography were considered to be not useful in the majority of the cases (91% and 74%, respectively) at the time of IMPA diagnosis.

The diagnostic imaging findings therefore did not affect the overall case management at initial presentation, including no effects towards treatment interventions, in the majority of the dogs with IMPA in this study population. Currently, it remains routine to perform thoracic and abdominal imaging as part of the initial diagnostic investigations for canine IMPA. The primary purpose of such imaging is to help assess for possible disease processes that may be associated with an IMPA. The use of diagnostic imaging also raises the matter of optimising resources and accompanying patient risks. For example, the availability of diagnostic imaging specialists (particularly in the context of abdominal ultrasound in general practice), anaesthesia specialists (such as if patients present as markedly moribund and require sedation or general anaesthesia for investigations), radiation risks to patients and personnel, and risks involved with sedation and general anaesthesia in ill patients.

Similar findings regarding the lower utility of thoracic and abdominal diagnostic imaging have been documented in previous studies investigating canine immune-mediated haemolytic anaemia [20,21], and dogs with fever diagnosed with non-infectious inflammatory diseases [22]. The findings from this study and the aforementioned studies thus further augment the potential relevance of future investigations regarding the utility of diagnostic imaging as part of the initial workup of other suspected immune-mediated diseases in small animals.

More agreement was found between the internists when assessing the thoracic radiography results, with full agreement in 95% of the cases. When assessing abdominal ultrasonography, full agreement was still made in the majority of the cases, but only in 61% of them. In addition, no full disagreements were made in the thoracic radiography, suggesting that the internists’ opinions were more similar when reading thoracic radiography. Conversely, in the abdominal ultrasonography, there were cases of partial disagreement and full disagreement where some clinicians gave a score of 2 whilst others gave a score of 4. The difference in agreement between thoracic radiography and abdominal ultrasonography is potentially due to the increased number of organs assessed in abdominal ultrasonography. Thus, abdominal ultrasound inherently has a higher chance to yield more findings with variable possible interpretations, particularly if the findings are non-specific or considered mild. This was evident in the fact that there were no cases of full disagreement in thoracic radiography, whilst there were two cases in abdominal ultrasonography.

Additionally, 89% of the dogs with significant abnormalities noted on abdominal ultrasonography, who therefore received a majority score of 4 by the reviewing internists, underwent further investigations at the actual time of presentation. In comparison, only 33% of the dogs with similarly significant abnormalities noted on thoracic radiography underwent further investigations. This may be due to the logistical factors associated with each imaging modality. Abdominal ultrasonography reveals real-time findings and is performed by board-certified diagnostic imagers or supervised diagnostic imaging residents at the referral hospital from which this study has originated from. Thus, this can positively impact the ability to decide and perform further investigations and sampling during ultrasound and is overall logistically more convenient to do so than in radiography. Conversely, thoracic radiography is often not performed by a diagnostic imager (board-certified or resident-in-training) at the stated referral hospital, and therefore, there is typically a longer interval in radiography reporting. This may therefore affect a clinician’s decision making to perform further investigations. For example, if a patient has completed its diagnostic imaging and would then require repeat sedation or anaesthesia for further investigations. In addition, investigations of the lower airways, such as a BAL, can be more logistically challenging than performing FNA in ultrasound. These factors may therefore have influenced the fewer patients undergoing further investigations following abnormal findings on thoracic radiography in this study population.

One must bear in mind that all scoring was based on the retrospective assessment of diagnostic imaging reports, and performed by internists who were not involved in the actual management of the cases. The specific decision to perform further investigations was made by the attending clinician of each case at the actual time of initial case presentation, and therein lies the limitation of the retrospective study design. It was beyond the scope of this study to assess the results and significance of the further tests performed in every case and therefore, the authors made the decision to focus on cases that received a majority score of 4.

Overall, the most common abnormal findings on thoracic radiography were considered mild and non-specific [13,14] in the majority of the cases and did not appear to significantly affect the overall case management at the time of IMPA diagnosis. The most common abnormal findings on abdominal ultrasonography were again relatively non-specific [15,16,17]. The authors considered the common findings of lymphadenomegaly, peritoneal effusion, and splenomegaly most likely secondary and in reaction to the patients’ underlying immune-mediated polyarthritis, given the absence of otherwise significant findings for the majority of cases who underwent sampling. Similar non-specific sonographic findings have also been documented in previous studies of canine IMPA [11,23].

Regarding joint radiography, erosive changes were documented in a small minority of cases [2% of the total study population], similar to what has been previously described [4,24]. The most common joint radiographed in this study population was the carpus, which also reflects what has been found in previous studies [7,23]. However, further investigation into the utility of joint radiography in canine IMPA was beyond the scope of this study.

From thoracic radiography, abnormal findings that were considered to immediately impact management of the patient’s IMPA (score 4) were only identified in a small number of the cases. Thus, it is possible that the abnormal findings were either detected very early on in the course of the respiratory disease (whether directly or indirectly related to the IMPA and patient’s systemic illness), or indeed that the radiographic changes were not significantly associated with the development of IMPA itself, as none of these cases (six dogs) had respiratory clinical signs at initial presentation.

In abdominal ultrasonography, a total of nine cases had significant abnormalities considered (majority score of 4). Of these nine cases, two presented initially with gastrointestinal signs (one with dysphagia and one with diarrhoea). One of these nine cases presented with haematuria and was diagnosed with a urinary tract infection on urine analysis obtained via cystocentesis (urine analysis performed as standard investigations for IMPA). The abnormal findings documented in the seven cases who underwent further sampling were predominantly noted in the lymph nodes (lymphadenomegaly), spleen (splenomegaly), and liver (hepatomegaly and altered parenchymal echogenicity). In the majority of these cases, cytology was most compatible with the inflammatory changes [25] and thus may reflect the response to the patient’s underlying IMPA.

As previously discussed, mild to moderate lymphadenomegaly was in the authors’ opinion most likely related to the patient’s generalised inflammatory response in the face of IMPA. However, as the sonographic imaging of lymph nodes is not specific to differentiate reactive change from malignancy [26,27], to confirm this hypothesis, every enlarged lymph node would require aspiration with adequate cytology for assessment. Therefore, as not all enlarged lymph nodes were aspirated, this is a limitation of the study and the authors’ opinion. In a similar fashion, no patients with a peritoneal effusion identified on ultrasound underwent sampling of the effusion. Hence, the exact nature of the effusions remained unknown in this population. This limitation was influenced by the reported small volumes of effusion in each case, and considered not amenable for sampling by the operating diagnostic imager.

On the other hand, one dog had a possible hepatic mass and hepatic nodules, and one dog had a duodenal mass. Cytology from the first dog’s liver and the second’s duodenal mass did not reveal any significant abnormalities at that time point, and therefore, sampling did not impact the immediate case management. However, one limitation is that in the dog with a duodenal mass, the FNA cytology results were non-diagnostic and therefore, the true aetiology of this mass in relation to its IMPA remained elusive.

The main limitations of this study include its retrospective study design, and that the eventual further diagnostic investigations and management of each case were at the discretion of the attending clinician. In addition, in the patients who presented with overt signs of neoplasia such as lymphoma (for example with generalised marked lymphadenomegaly), or in the patients who presented with concurrent joint and spinal pain and thus raised concerns of a steroid-responsive meningitis-arteritis (where concurrent IMPA can occur) [28], it is possible that arthrocentesis was foregone in these cases. Therefore, these dogs would not be captured in our study population. Additionally, certain abnormal findings on diagnostic imaging such as pulmonary patterns and lymphadenomegaly inherently have marked limitations. The severity of such findings is often based on subjective assessment and interpretation by the reporting diagnostic imager, and therefore, can subsequently affect interpretation by internal medicine clinicians. However, for the purpose of this study, the authors considered it important to categorise the severity of such findings (pulmonary patterns and lymphadenomegaly), as they could affect the internists’ analysis and scoring of the utility of thoracic radiography and abdominal ultrasonography overall.

Another considerable modality for diagnostic imaging in dogs with suspected IMPA includes computed tomography (CT). As this advanced modality may have revealed more pertinent findings, particularly with regard to pulmonary assessment, it is possible that additional significant findings were not observed on plain radiography in this population. However, given the financial factor and general anaesthesia requirements to perform CT at this referral hospital, CT is typically reserved for cases with significant respiratory clinical signs. As such clinical signs were not observed in this study cohort, no CT reports were available for analysis in the study population. In addition, plain radiography is considered a more common and available imaging modality in the majority of veterinary clinics and hospitals. Thus, for the purpose of this study, it was elected to assess only plain radiography. As this study was performed at a single UK referral hospital between 2008 and 2012, infectious disease testing was performed based on geographical location and endemic infectious diseases of the UK at the time. No included case was known to have travelled overseas, and therefore, additional infectious disease testing for pathogens such as fungi and leishmaniasis were not performed. However, in the current face of increasing pet travel and climate shift, veterinarians should certainly consider a wider array of infectious testing in the future, in accordance with patient history. Finally, it is extremely challenging and often not feasible to definitively prove whether a disease process documented during investigations for IMPA is a solely unrelated comorbidity of the patient, or whether it is associated with the IMPA itself. On the other hand, in the clinical setting, if a significant disease process is identified, at the attending clinician’s discretion, it is often considered most likely an associated disease (such as type II or type III IMPA).

Future studies assessing the utility of diagnostic imaging in canine IMPA that include a larger sample population and larger number of small animal internal medicine specialists would be beneficial to further support and strengthen the findings from this descriptive study. It would also be of interest to investigate how presenting clinical signs, patient signalment, haematology, serum biochemistry, and urine analysis results may correlate with diagnostic imaging findings, and how they may affect decisions for the diagnostic imaging of the thorax, abdomen, and joints, as well as decisions for additional investigations. In addition, the utility of further investigations performed based on diagnostic imaging findings, such as cytology and cultures, on the overall case management of dogs with IMPA may also be of interest to investigate in the future, as well as the utility of joint radiography. Finally, as this study assessed diagnostic imaging at the time of the initial diagnosis of IMPA, it may be of relevance to study the utility of thoracic, abdominal, and joint imaging in cases of canine IMPA that relapse or that do not respond well to standard therapy, particularly as progression from apparently non-erosive IMPA to erosive polyarthritis is possible [9].

## 5. Conclusions

In conclusion, the results of this study revealed that although both thoracic radiography and abdominal ultrasonography identified numerous abnormal findings, the findings were deemed not useful in the overall case management for the majority of the dogs at the time of IMPA diagnosis. Therefore, judicious consideration should be taken when deciding on, and discussing with owners, the costs and benefits of performing thoracic radiography and abdominal ultrasonography as part of the initial diagnostic investigations for canine IMPA.

## Figures and Tables

**Table 1:** Classification (type) of immune-mediated non-erosive polyarthropathies [1].

Type	Description
Type I (idiopathic)	No associated disease documented
Type II (reactive)	Associated disease documented—infectious or inflammatory disease distant from the joint
Type III (enteropathic)	Associated disease documented—gastrointestinal or hepatic disease
Type IV (neoplastic)	Associated disease documented—neoplasia distant from the joint

**Table 2:** Clinical signs reported at initial presentation.

Clinical Sign	Number of Cases
Lameness	42
Joint swelling	38
Pyrexia	30
Lethargy	27
Joint pain	20
Stiff gait	15
Hyporexia	14
Diarrhoea	7
Peripheral lymphadenomegaly	7
Vomiting	6
Cough	2
Dermatitis	2
Dysphagia	2
Haematuria	2
Joint thickening	2
Non-specific pain	2
Spinal pain	2
Ataxia	1
Aural abscess	1
Collapse	1
Abdominal pain	1
Peripheral oedema	1
Weight loss	1

**Table 3:** Findings on thoracic radiography.

Finding	Number of Cases
Bronchial pattern	
Mild	8
Bronchointerstitial pattern	
Mild	15
Moderate	5
Marked	2
Interstitial pattern	
Mild	9
Moderate	2
Marked	1
Alveolar pattern	
Moderate	1
Increased pulmonary opacity	1
Sternal lymphadenomegaly	
Mild	1
Cardiac silhouette size	
Reduced	1
Enlarged	5
Pleural effusion	
Mild	1
Pleural fissure lines	2
Pneumoperitoneum and pneumoretroperitoneum	1

**Table 4:** Overall Diagnostic Utility (ODU) for thoracic radiography.

	Full Agreement (Cases)		Partial Agreement (Cases)	
Thoracic radiography	73/77		4/77	
ODU	Useful	Not useful	Useful (majority)	Not useful (majority)
	5/73	68/73	2/4	2/4

**Table 5:** Diagnostic Utility Score (DUS) for thoracic radiography.

	Full Agreement (Cases)				Partial Agreement (Cases)			Full Disagreement
Thoracic radiography	65/77				12/77			0/77
DUS	Score 1	Score 2	Score 3	Score 4	Score 2	Score 3	Score 4	-
	30/65	30/65	0/65	5/65	8/12	3/12	1/12	-

**Table 6:** Findings on abdominal ultrasonography.

Finding	Number of Cases
Lymphadenomegaly	
Mild	43
Moderate	4
Marked	1
Peritoneum	
Peritoneal effusion	11
Spleen	
Splenomegaly	11
Splenic nodules	2
Heterogeneous echogenicity (mixed)	2
Liver	
Hepatomegaly	8
Hepatic mass/nodule	1
Hyperechoic echogenicity	1
Hypoechoic echogenicity	1
Heterogeneous echogenicity (mixed)	2
Biliary tract	
Common bile duct distension	1
Thickened gall bladder wall	2
Thinned and hyperechoic gall bladder wall	1
Gall bladder sludge	5
Pancreas	
Chronic pancreatopathy	3
Gastrointestinal tract	
Gastroenteritis-colitis	8
Gastrointestinal foreign body	2
Kidneys	
Chronic nephropathy	8
Urolithiasis	3
Pyelectasia	2
Renomegaly	1
Adrenal glands	
Adrenomegaly	1
Urinary bladder	
Polypoid wall lesion (compatible with recent cystotomy site)	1
Urinary sediment	3
Urinary bladder wall thickening	1
Reproductive tract	
Benign prostatic hypertrophy	6
Prostatitis	1
Cystic endometrial hyperplasia and fluid within uterine lumen	1

**Table 7:** Overall Diagnostic Utility (ODU) for abdominal ultrasonography.

	Full Agreement (Cases)		Partial Agreement (Cases)	
Abdominal ultrasonography	47/77		30/77	
ODU	Useful	Not useful	Useful (majority)	Not useful (majority)
	4/47	43/47	16/30	14/30

**Table 8:** Diagnostic Utility Score (DUS) for abdominal ultrasonography.

	Full Agreement (Cases)				Partial Agreement (Cases)			Full Disagreement
Abdominal ultrasonography	46/77				29/77			2/77
DUS	Score 1	Score 2	Score 3	Score 4	Score 2	Score 3	Score 4	-
	6/46	33/46	5/46	2/46	12/29	9/29	8/29	-

## Data Availability

The data are contained within the article. The data presented in this study are available in the tables and figures in the article “Diagnostic utility of thoracic radiography and abdominal ultrasonography in canine immune-mediated polyarthritis: 77 cases”.
